# Experimental Phenomenology as an Approach to the Study of Contemplative Practices

**DOI:** 10.3389/fpsyg.2021.751298

**Published:** 2022-01-10

**Authors:** Lars-Gunnar Lundh

**Affiliations:** Department of Psychology, Lund University, Lund, Sweden

**Keywords:** experimental phenomenology, contemplative practices, mindfulness, gratitude, receiving, giving, embodiment, mindful driving

## Abstract

During history humans have developed a large variety of contemplative practices, in many different areas of life, and as part of many different traditions and contexts. Although some contemplative practices are very old, the research field of Contemplation Studies is young, and there are no agreed-upon definitions of central concepts such as contemplative practices and contemplative experiences. The present paper focuses on contemplative practices, defined as practices that are engaged in for the sake of the contemplative experiences they afford (e.g., the contemplation of nature, or the contemplation of various aspects of being-in-the world). The purpose of the present paper is to discuss the potential of experimental phenomenology to contribute to the development of the research field of Contemplation Studies. Experimental phenomenology is defined as the investigation of phenomenological practices and their effects on experience. Phenomenological practices involve intentional variations of experiencing by means of changes in the direction of attention and the choice of attitude, typically as guided by verbal instructions or self-instructions. It is suggested that contemplative practices represent a subcategory of phenomenological practices. Two different varieties of experimental phenomenology are described and illustrated in the present paper: (1) an informal variety which involves the development of new phenomenological practices by creative variation of procedures and observation of effects; and (2) a more rigorously scientific variety, which involves the systematic variation of phenomenological practices in accordance with experimental designs to study their experiential effects. It is suggested that the development of contemplative practices during the ages is the result of an informal experimenting of the first kind; this variety of experimental phenomenology can also be used to develop personalized health interventions in a clinical setting. As to the more rigorously scientific experimental phenomenology, it is possible that it may lead not only to an improved understanding of general principles underlying contemplative practices, but also to a more systematic development of new contemplative practices. The experimental-phenomenological approach to contemplative practices is illustrated by various examples involving mindfulness, gratitude, receiving and giving.

## Introduction

The word contemplation has been defined in different ways (e.g., Komjathy, 2017), but most basically it refers to a certain form of attention and reflection, which is illustrated by some examples provided by the Merriam-Webster Dictionary (n.d.). Among these examples of how the word is used are (1) that a person makes a decision “after much contemplation,” (2) is “lost in quiet contemplation of a scene,” and (3) “goes to the forest to spend time in contemplation of nature.” Of these three examples, the first exemplifies a highly instrumental form of contemplation where there is no need for any contemplative experience – the intention is simply to contemplate the consequences of various alternatives to take a decision. In the second example, there is mention of a *contemplative experience* (i.e., an experience with contemplative qualities), but not necessarily as the result of an intentionally undertaken contemplative practice – the example simply speaks of a person who gets “lost in quiet contemplation of a scene,” which may happen quite unintentionally. In the third example, however, we find what may be called a *contemplative practice*. A person who goes to the forest to spend time in contemplation of nature can be said to engage in contemplation for no other purpose than the contemplative experience itself. In this paper, I will use this as a paradigm example of a contemplative practice, and thereby propose a first preliminary working definition of contemplative practices, which, however, may need to be revised or elaborated as the result of further considerations: *Contemplative practices are practices which are engaged in for the contemplative experiences that they afford*.

In this perspective, a contemplative experience is seen as valuable as such, whether it has any other further consequences or not. One implication, for example, is that contemplative experiences may be of considerable potential value even for a dying person, who has no future (and where future consequences are nonexistent). In terms of psychological theory, when a person chooses to engage in a contemplative practice for no other purpose than the contemplative experience itself this is an example of *intrinsic* rather extrinsic motivation. Intrinsic motivation is defined as doing something because it is inherently interesting or enjoyable or satisfying (e.g., Ryan and Deci, 2000), rather than as a means for reaching some other kind of goal.

Another more instrumental perspective on contemplative practices is to see them as serving a higher purpose than merely the contemplative experience itself. This is the position taken, for example, by Komjathy (2017) when he states that, “Contemplative practice refers to various approaches, disciplines and methods for developing attentiveness, awareness, compassion, concentration, presence, wisdom, and the like.” (p. 68). In this perspective, contemplative practice is engaged in for the purpose of some form of self-improvement. At the same time Komjathy (2017) is very open regarding the definition of contemplative practices, stating that from his perspective “definitional parameters should be explored and discussed, rather than rigidly defined” (p. 69).

It may be argued that these two perspectives are complementary. Even if engaging in a contemplative practice may be intrinsically motivating because of the contemplative experiences that it affords in the moment, this is quite compatible with the assumption that a *regular* engagement in contemplative practices may have long-term consequences in the form of increased attentiveness, presence, awareness, empathy, compassion, wisdom, and other virtues. And if so, this may be sufficient reason to develop a long-term commitment to engage in contemplative practices. This is also consistent with meta-analyses and systematic reviews of existing research which suggest that attention-based forms of meditation may have effects on at least some aspects of attentional processes (Sumantry and Stewart, 2021; Yakobi et al., 2021), and that meditation (e.g., compassion-based meditation) may lead to increased empathy, compassion, and prosocial behaviors (Luberto et al., 2018).

Komjathy (2017) describes three characteristics of contemplative practice: (1) a commitment to practice; (2) a focus on personal experience from a first-person perspective (which he labels “critical subjectivity”); and (3) an ambition for personal “character development” (p. 36). Of these, it may be argued that a focus on personal experience from a first-person perspective is the most basic and essential one. For example, it is quite possible to engage in contemplative practices (e.g., going to the forest to spend time in contemplation of nature) on an irregular basis, without any commitment to practice and without any ambition for “character development.” But it is hardly possible to engage in a contemplative practice without having a focus on one’s present experience.

From an instrumental perspective on practicing, it is of course possible to disregard one’s present experience in the service of some future goal. As stated by Komjathy (2017) in his chapter on contemplative experiences, “I believe that contemplative practice is and should be primary… One reason for this is because committed and prolonged contemplative practice often involves ignoring ‘experience’ or overcoming certain experiential patterns. That is, on a deeper level, contemplative practice often requires perseverance in the face of difficulties or in the absence of ‘benefits.’ One must sometimes set aside ‘experience’ in the name of ‘practice.”’ (p. 105). This, however, raises the question whether a contemplative practice without contemplative experiences is still in fact a contemplative practice, or if it has now turned into something else.

At the very least, these considerations suggest that we may perhaps need to differentiate between single contemplative practices, and contemplative practice as a “life project” – which Komjathy (2017) describes as a “contemplative approach” to life, or as “following a contemplative path” (p. 72). One further implication of such a differentiation is that contemplative practice as a life project may well involve many other activities besides typical contemplative practices. As described by Komjathy (2017), contemplative practice represents “an approach that may be applied to and expressed in almost any activity” (p. 35), including art, dancing, writing, photography, etc.

From a developmental perspective it might be of interest to use longitudinal research designs to study the variety of “contemplative paths” that can be seen in different individuals. One type of path may involve an individual who starts out by exploring various contemplative practices for the sake of the contemplative experiences that they afford in the moment, and later develops a personal commitment to contemplative practice that leads to the development of new skills and various forms of character development. Another possible type of path might involve less benign developmental processes, where an initial openness to explore contemplative practices is followed by setbacks in the form of “dark nights and spiritual emergencies” (Komjathy, 2017, p. 122). Although the research on positive and negative experiences in connection with meditation has expanded during later years it still rests on cross-sectional surveys and interviews (e.g., Lindahl et al., 2017; Vieten et al., 2018). A next more challenging task would be to use longitudinal research designs to study positive and negative developments among practitioners over the years.

During history humans have developed a large variety of contemplative practices. Much of this development originally occurred as part of various religious traditions, but more recently we have also seen the development of many contemplative practices in secular contexts. A recent development is what Komjathy (2017) has described as the emergence of a more comprehensive interdisciplinary field of Contemplation Studies. Although he describes this field as “diverse, decentralized, and experimental” (p. 4), without any dominant model or paradigm, he also discerns “some influential expressions, recurring patterns, and emerging trends” (p. 4). At the same time, he emphasizes the need for “a comprehensive, representative, and integrated discussion of the field, including a ‘generous reading’ and ‘critical evaluation’ of the contributions and limitations of its various expressions” (Komjathy, 2017, p. 4).

Some well-known examples of contemplative practices in the Western world today are mindfulness meditation and yoga; surveys indicate an increasing use of yoga and meditation during the last decades, among both adults and children (e.g., Cramer et al., 2016; Wang et al., 2019). Among the many other examples of contemplative practices that are mentioned by Komjathy (2017) in his overview are Tai Chi, Qi Gong, and various forms of contemplative prayer, as well as more recent additions to the field such as Alexander technique (Alexander, 1932), the Feldenkrais method (Feldenkrais, 1972), and Gendlin’s (1978) Focusing.

In the present paper the focus lies on the specific kind of attention and attitude to one’s experiences that is involved in contemplative practices. This is approached from a phenomenological perspective. More specifically, it is approached from a perspective that has previously been described as experimental phenomenology (Lundh, 2020) and has its roots in Husserl’s (1938/1970) philosophical phenomenology and Ihde’s (1977, 2012) experimental phenomenology.

A rigorous scientific study of contemplative practices requires a comprehensive approach in terms of an integrative conceptual framework. The purpose of the present paper is to discuss the potential of experimental phenomenology to contribute to the development of such an integrative framework. The paper has two main parts. The first part of the paper describes the conceptual framework of experimental phenomenology and gives examples of how experimental phenomenology can be used both to develop new phenomenological practices and to study existing ones. In the second part, various kinds of contemplative practices are discussed from the perspective of experimental phenomenology. To enable a certain breadth in the discussion, examples are taken from widely different traditions.

## Experimental Phenomenology

The purpose of this section is to describe the basic conceptual framework of experimental phenomenology and to say something about its relation to theoretical and descriptive forms of phenomenology. As phenomenology is a vast and many-faceted area, however, the relation between experimental phenomenology and other varieties of phenomenology is only dealt with very briefly here; for more detail the interested reader is referred to Lundh (2020). It should also be noted from the start that although experimental phenomenology is influenced by Husserl’s (1938/1970) philosophical phenomenology, it makes no claim to follow Husserl’s philosophy in any orthodox manner.

Phenomenology can be defined as the scientific study of our experience of being in the world. According to Husserl (1938/1970), doing phenomenology means to turn our attention to experience as such. This represents a shift in perspective from our usual *natural attitude* with its focus on the world (and our practical engagement with things in the world) to a *phenomenological attitude* characterized by a focus on the experience of being in the world. Instead of focusing on the objective world as it appears *from* our subjective perspective (i.e., the natural attitude), attention is turned *to* the subjective perspective as such (a phenomenological attitude).

Experimental phenomenology is a subvariety of phenomenology, which differs from theoretical and descriptive varieties of phenomenology by involving the investigation of phenomenological *practices* and their impact on subsequent experience (Lundh, 2020). The task of experimental phenomenology, as it is understood here, is (1) to increase our understanding of phenomenological practices by a systematic variation of these and a study of their effects, and (2) to develop new phenomenological practices that can have a beneficial influence on people’s life quality and personal development.

It is important to note that phenomenology in this sense, including experimental phenomenology, is not some kind of “first-person science.” As pointed out by Thompson (2020, p. 46) among others, “Although Husserl’s phenomenology has occasionally been misdescribed as an effort to do ‘first-person-science,’ he didn’t describe it this way, but rather presented it as a collective and intersubjective project” (p. 46-47). This is also true of experimental phenomenology, as emphasized in a previous paper:

“What makes experimental phenomenology into a scientific endeavor is the intersubjective nature of this kind of study: potential effects described by one person can be subjected to replication both by the same person, and by other persons. Also, conclusions drawn on the basis of this kind of study are hypothetical and provisional and may be modified or specified on the basis of further study” (Lundh, 2020, p. 493).

The first researcher to speak of an experimental phenomenology was probably Don Ihde (1977), although in a different context than the present one – the study of perceptual illusions (e.g., the Necker cube) and how intentional variations of subjective experiencing can affect our experience of these illusions. What was new about Ihde’s (1977) approach was that it was designed to “show how to *do* phenomenology as a *praxis*” (Ihde, 2012, p xii). Since then the term “experimental phenomenology” has also been used by other writers in a quite different sense, as for example by Albertazzi (2019) who defines experimental phenomenology simply as “the study of appearances in subjective awareness” (p. S2191), without any mention of phenomenological practices. As experimental phenomenology is understood here, however, it involves *an active experimentation* with one’s subjective direction of attention and choice of attitude.

### Phenomenological Practices

As defined here, phenomenological practices involve an intentional variation of subjective experiencing by changes in the direction of attention and the choice of attitude. Typically, this is guided by some form of verbal instructions and self-instructions; these instructions are what differentiate one phenomenological practice from another. To engage in a phenomenological practice is, by definition, to focus attention on one’s experiences with some specific kind of attitude, according to some set of instructions.

These instructions may be given by another person (e.g., a teacher or a therapist), or they may be given by the experiencing person in the form of self-instructions, but they are always about one’s attention and attitude. To follow this set of instructions means to deliberately modify one’s direction of attention and/or one’s attitude in a specific way. The content of these instructions is what defines the nature of each specific phenomenological practice. This also means that *verbal precision* is one of the priorities in experimental phenomenology. Different words carry different meanings, and verbal modifications often imply modifications in the phenomenological practice involved.

One example of a phenomenological practice is mindfulness meditation. Although there are many definitions of mindfulness in the literature, most of them mention at least two basic instructions: (1) to deliberately focus attention on some aspect of present experience; and (2) to do this with a particular kind of attitude, characterized as being accepting and non-judging; kind, friendly and caring; and showing openness, curiosity, and non-reactivity to experience (e.g., Bishop et al., 2004; Kabat-Zinn, 2013; Shapiro and Carlson, 2017). As a corollary, this also implies an instruction to gently bring attention back to the present moment when getting distracted by thoughts. All these instructions are about regulating our inner experiencing, and do not have to involve any publicly observable behavior.

Although phenomenological practices *need* not involve any change in observable behavior, however, some practices do. Although these practices are not *purely* phenomenological, they still are phenomenological practices to the extent that they involve instructions concerning attention and attitude. In a previous paper, this was illustrated by a comparison between different breathing exercises:

“A breathing meditation that simply involves attending to one’s breathing just as it is, with no instruction to change it in any way, is a pure phenomenological practice. A breathing exercise where the individual is instructed to breathe in a particular way (for example, slowly or deeply), while at the same time paying close attention to the breathing, is not a pure phenomenological exercise because it also involves a change in overt behavior. It is still a phenomenological practice, however, because it essentially involves instructions about the direction of attention. On the other hand, if the instructions are only about breathing in a particular way, and do not say anything about attention or attitudes, it is not a phenomenological practice but a pure behavioral practice” (Lundh, 2020, p. 497–498).

One aspect of phenomenological practices is that they represent techniques that are possible to formulate in words and to describe either orally or in writing. This makes it possible to *teach* phenomenological practices. Another aspect of phenomenological practices, however, is that they cannot be *reduced* to the application of techniques, or pure routines, without losing their nature as phenomenological practices. Routinely practiced exercises may well have their value, but without an element of consciously focused attention on the practice while it is engaged in it does not count as a phenomenological practice. As emphasized by Ellen Langer (2014), such consciously focused attention involves an openness to *new* experiences, and this openness to novelty is one thing that differentiates mindful from “mindless” activity.

#### Contemplative Practices as a Subcategory of Phenomenological Practices

Although all phenomenological practices involve the regulation of attention and attitudes, all phenomenological practices are not contemplative. One example is hypnosis, where instructions are typically given in the form of a hypnotic induction that is designed to affect the person’s attention or experiences in a specific way, while leaving little room for contemplation. This clearly represents a phenomenological practice, as defined here, in the sense that instructions of some form are given to subjects to make them modify their direction of attention and attitude. Although hypnosis has been defined in several different ways, one of the more cited definitions sees it as a “state of consciousness involving focused attention and reduced peripheral awareness characterized by an enhanced capacity for response to suggestion” (Elkins et al., 2015). Although this clearly implicates both attention and attitude (in the form of an increased suggestibility) there is nothing that implicates any contemplative experiences. It is also easy to imagine other examples of practices where attention and attitude are manipulated in some way without any contemplative purposes.

Even if all phenomenological practices are not contemplative, a working hypothesis may be that all contemplative practices are phenomenological, in the sense that they all involve instructions about the direction of attention and choice of attitude. If so, contemplative practices would represent a subcategory of phenomenological practices. But is this true? One difficulty here is the absence of an agreed-upon definition of what constitutes a contemplative practice (Komjathy, 2017). To answer this question we would have to be certain about which practices are to be characterized as contemplative, and this cannot be done without a clear definition of what makes them contemplative in the first place.

One way of solving this problem is by defining contemplative practices as phenomenological practices that are engaged in for the sake of the contemplative experience that they afford. This would be consistent with the reasoning in the introduction to the present paper. This would, for example, rule out a variety of phenomenological practices that are used in a psychotherapeutic context.

In classical psychoanalysis (Freud, 1916), for example, the analyst instructs the patient to use “free association,” which means to enter a state of quiet, unreflecting self-observation, and to report all associations that appear, trying not to censor anything. Here the instructions clearly involve both a change of attention (i.e., to attend to all kinds of associations that turn up) and of attitude (i.e., taking a non-defensive attitude to potentially disturbing associations), and thereby clearly qualifies as a phenomenological practice. Even *if* this would also result in contemplative experiences now and then, it would not be classified as a contemplative practice because it is not undertaken for the sake of the contemplative experience as such, but rather in the service of goals such as psychoanalytic insight and personal development. On the other hand, someone might argue – along similar lines as Komjathy (2017) does with regard to contemplative practice as a life project – that even if psychoanalysis is not undertaken for the sake of contemplative *experiences*, it might represent a contemplative *path* that can lead to “character development.” Because the present paper focuses on single contemplative practices, rather than on contemplative practice as a life project, however, this question need not bother us further here. But it illustrates some of the definitional problems that are at issue.

Consider now the case of meditation. Meditation is often seen as the most prototypical form of contemplative practice (cf. Komjathy, 2017). But there are different forms of meditation. Are they all to be seen as phenomenological practices? In other words, do they all involve instructions about attention and attitude? Dahl et al. (2015) have proposed a classification system that categorizes specific styles of meditation into attentional, constructive, and deconstructive families. According to their classificatory scheme, mindfulness meditation belongs to the “attentional” category, whereas lovingkindness meditation belongs to the “constructive” category, and various forms of insight meditation belong to the “deconstructive family.” At first sight, the very naming of these categories might suggest that only the first category involves instructions about the direction of attention. However, “attentional” in Dahl et al.’s (2015) scheme refers to the hypothetical “cognitive mechanism” that is supposed to be involved, rather than to the contents of the instructions given. Looking at the verbal instructions in lovingkindness meditation (e.g., Salzberg, 2002), these clearly speak explicitly both about direction of attention (first to specific individuals and then eventually to all beings) and about a very specific attitude (described in terms of kindness and compassion).

This also illustrates an important thing about experimental phenomenology: Experimental phenomenology is interested primarily in the specific *content* of contemplative practices, as described in the verbal instructions given, and not in explanatory mechanisms. The latter are the subject of experimental cognitive psychology and neuropsychology, rather than phenomenology. This does in no way detract from the importance of also studying questions about mechanisms, but these questions simply do not belong to the field of experimental phenomenology.

### Intentional Variation of Phenomenological Practices

Experimental phenomenology as defined here involves an intentional *variation* of phenomenological practices. This includes a variation in the direction of attention and/or the choice of attitude, as guided by some set of verbal instructions or self-instructions. Such an intentional variation of phenomenological practices may take place, for example, in a therapeutic context (e.g., the development of personalized health interventions; Lundh, 2021) or in a research context (i.e., to compare different varieties of phenomenological practices as to their effects; Lundh, 2020). In other words, experimental phenomenology basically rests on an active, intentional variation of phenomenological practices, either for the purpose of developing new practices that are suited to a specific person, or for the purpose of studying the effects of these practices. In this section, these two different applications of experimental phenomenology are described and illustrated by examples.

#### Experimental Phenomenology as a Personalized Health Intervention

Experimental phenomenology may be used to construct personalized health interventions. One example (described by Lundh, 2020) is the construction of a mindful driving practice for a client who had problems with sleepiness while driving. In this case specific personalized instructions for mindful attention were developed successively and tested while driving shorter distances. The result was the construction of a mindful driving practice where the individual shifted, in accordance with the changing traffic conditions, between four different phases of mindful attention, each with a specific focus: the road, the traffic, the landscape, and the sitting. The client was instructed to regulate attention by shifting in a smooth way between these four phases, according to principles of second-order mindfulness (Langer, 2014). Importantly, the client in question participated actively in shaping the details of the mindful driving instructions, so that they would suit his needs and preferences and have desirable effects in this specific case.

Another example is a mindful embodiment meditation that was developed for a client with insomnia due to early morning awakenings (Lundh, 2021). A set of detailed self-instructions was developed in collaboration with the client, designed to express (1) an explorative attitude toward the body, with (2) breathing as a central component, and (3) proceeding slowly. Four parts of the body were identified where the client felt especially tense, and which were focused during the meditation: the eyes, the lips, the neck, and the chest. After having learned the basic set of self-instructions the client was also encouraged to shift freely between the different components of the meditation, in accordance with principles of second-order mindfulness (Langer, 2014), thereby leaving room for improvisation and playfulness within the given frame of the practice.

It should be noted that these examples do not represent experimental phenomenology as a scientific method, but rather as a clinically useful strategy to develop an individually tailored health intervention. The term “experimental” is still adequate, although here it refers to a looser form of experimenting than that involved in scientific research. The latter more rigorous kind of experimental phenomenology is described in the next section.

Before leaving the topic of personalized health interventions it may be asked whether “mindful driving” and “mindful embodiment” could be characterized as contemplative practices. Here it is relevant to note that the client who practiced mindful driving noted a new and more positive experience in the sense that “he could now *enjoy* driving in a new way, as it afforded him new visual and other sensory impressions of the road, the surrounding landscape, and the traffic flow” (Lundh, 2020, p. 501). Similarly, the client who practiced mindful embodiment reported using it also in situations that were usually experienced as “boring,” such as waiting for the bus or sitting in a waiting-room, and noted that the situation no longer felt boring. *If* contemplative practices are defined, as suggested above, in terms of the inherently rewarding contemplative experiences that they afford, the practices in question may well be characterized as contemplative.

#### Experimental Phenomenology in a Research Context

Consider again the mindful embodiment meditation that was mentioned in the previous section. What would it take to study this in terms of a more rigorous experimental approach? As outlined in a previous paper (Lundh, 2020), such an undertaking would require a *systematic variation* of the phenomenological practice itself. And what may be varied here is either the direction of attention or the attitude. In this specific practice, attention was directed to four different parts of the body (the eyes, the lips, the neck, and the chest), and the instructions contained three different attitudes that were taken successively (exploration, “breathing into,” and proceeding slowly). One way to conduct a more rigorous exploration of the effects of this phenomenological practice is to systematically vary these elements according to an experimental design.

The matrix described in Table 1 illustrates the various combinations of attention and attitude that were used for a similar purpose in an unpublished pilot study. Here two additional attitudes were entered, which both implicated a loving attitude. In the verbal instructions given, the attitudes entered in relation to each of four bodily regions were expressed in the following form: “May I explore …,” “May I breathe into …,” “…slowly …,” “May I feel love for …, and “May I feel love in ….”

**TABLE 1:** The various combinations of attention (as described in columns) and attitude (as described in rows) that were used in a study with an experimental-phenomenological design.

	The eyes	The mouth	The neck/back	The chest
Explore				
Breathe into				
Go slowly				
Feel love for				
Feel love in				

In a first part of this study, the participants (who had previous experience with meditation) were asked to attend to their resulting experiences and to note these verbally on a form that was very similar to Table 1. A thematic analysis of these notes suggested that the five different attitudes tended to give different kinds of experiences. For example, the explorative attitude was associated with an increased awareness of bodily sensations in the attended region of the body, whereas the breathing-into instruction tended to be more associated with feelings of relaxation. There was also a differentiation in effect between the two loving attitude instructions. For example, whereas the instruction “May I feel love for my eyes” tended to be followed by feelings of gratitude for having eyes to see with, the instruction “May I feel love in my eyes” tended to be followed by feelings of warmth and kindness.

In a second part of this study the aim was to develop a questionnaire, with items based on the various responses reported in the first qualitative part of the study, and with a Likert-type response format to allow quantification of participants’ experiences. This represents a phenomenological mixed methods approach (cf. Martiny et al., 2021) which combines qualitative and quantitative methods. In the first part of the study, qualitative methods with an open response format were used to generate answers from the participants about their experiences – this is of particular importance in areas where it is unknown or uncertain which kind of experiences are to be looked for. This may then generate hypotheses about the effects of different kinds of attitudes and different directions of attention, that can be tested by quantitative methods. To increase the methodological quality of the first qualitative part of this kind of study it would also be possible to use microphenomenological interviews (Petitmengin et al., 2017; for a brief discussion of the role of micro-phenomenological methods in experimental phenomenological research, see also Lundh, 2020, p. 502).

As illustrated in this example, phenomenological practices may be varied systematically both as to the direction of attention and in terms of the attitude chosen. “Attitude” here refers to how one relates to the object of attention. In the above-mentioned example the objects of attention were four different regions of the body, and five different ways of relating to it were compared: by an explorative attitude, by breathing into it, by proceeding slowly, and by two different forms of a loving attitude. It may be questioned whether “breathing into” a certain part of the body really represents an attitude. It is possible that “attitude” is a too narrow term to use in this context, but for lack of a better term it is used here in a technical sense, as defined above (i.e., in terms of how one relates to the object of attention).

Another question that may be addressed experimentally is which impact a specific part of the practice has. For example, we might want to study if the instruction to proceed slowly has any specific effect, or whether it can be removed with no loss of effect. To go slowly here may be understood as the opposite of an intention to reach a goal as quickly as possible; here the intention rather expresses an attitude of patience and tranquility. One hypothesis is that proceeding slowly makes available more details to attend to and that it may thereby change the impact of a given practice. This also applies to the reading of verbal self-instructions. For example, a set of verbal instructions may be given more slowly, together with an (explicit or implicit) instruction to attend consciously to each word, while letting the meaning of each word “sink in.” The hypothesis that going slowly has these kinds of effects is possible to test by a variation in the instructions, and/or by a variation in the speed with which the instructions are read.

So far, what has been described as experimental phenomenology represents a systematic variation of *the phenomenological practice itself*. But one and the same phenomenological practice (as defined in terms of verbal instructions, direction of attention, and attitude) may have different impact also depending on the *context* and on the *person* who is engaging in it. For example, what tends to have a given impact for one person need not have the same impact for another person, and what has certain effects in one context need not have the same impact in another context. To address these questions, context and person may also be systematically varied.

Experimental phenomenology is first and foremost a *person-oriented* form of research (Bergman and Lundh, 2015; Lundh, 2015), where the purpose is primarily to draw conclusions at the level of the individual. Person-oriented research starts from the individual person and then generalizes from individual cases to larger groups of people and contexts. In this way it differs from *variable-oriented* research, which starts at the group level and conducts statistical analyses of differences between groups (e.g., an experimental group and a control group) and of correlations between different variables.

That is, although the primary purpose is to identify regularities in personal functioning, it is generally assumed that such regularities may differ from one individual to another, and from one context to another. This, however, in no way precludes the search also for regularities that generalize across individual persons, and across contexts. On the contrary, one aim of experimental phenomenology is to search precisely for such general principles. For example, as already suggested, one possible general principle might be that proceeding slowly during a phenomenological practice tends to have more beneficial effects than proceeding quickly.

#### Experimental Phenomenology and Experimental Group Comparisons

One way of further clarifying the nature of experimental phenomenology is by contrasting it with experimental group comparisons (i.e., a typical variable-oriented approach). As already explained, experimental phenomenology is *primarily* a person-oriented approach. It can, however, also make use of a variable-oriented approach by analyzing statistical differences in outcome between groups of participants who receive different sets of verbal instructions.

Langer et al. (2009, 2012) for example, have carried out a series of experimental group studies of mindfulness. To exemplify, in one study (Langer et al., 2009) orchestral musicians were divided into two groups, where those in the experimental group were asked to engage mindfully in their performance by actively noticing new things about the music they played, making new distinctions, and offering new subtle nuances while playing, whereas those in the control group were instructed to try to recreate a past performance of this piece of music. The results showed not only that the musicians in the experimental group enjoyed their performance more, and rated it as better, but also that the audience preferred the music that was created in a mindful state over music that was created by musicians who were merely trying to recreate a past performance. Although this was a study at the group level, where the effects were compared in terms of group averages, the study is an example of experimental phenomenology, because both the independent and dependent variables were operationalized phenomenologically (i.e., in terms of experiencing).

#### Experimental Phenomenology and Religious Approaches

Experimental phenomenology is a distinctly secular and psychological approach to contemplative practices, in the sense that it focuses on psychological aspects of contemplative practices such as the regulation of attention and attitudes, and that it aims to explore the effects of such practices. This, however, in no way means that it is restricted to studying contemplative practices of a secular origin. The characterization of experimental phenomenology as “distinctly secular and psychological” means that there are no limitations to what can be studied, as long as both the independent and dependent variables are phenomenological (i.e., refer to experiences). Thereby it is quite consistent, for example, with the search for “contemplative universals” in the mystical practices of different religions (e.g., Rose, 2016). It is clearly distinct, however, from those religious approaches where contemplative practices are used not only to explore human experience but also as a way of acquiring the beliefs that characterize the religion in question (which is sometimes labeled “insight”).

Experimental phenomenology is fully open to the possibility that at least for some individuals a given phenomenological practice may have the most beneficial effects when it is associated with various types of religious beliefs. The personalized focus of experimental phenomenology implies an openness to the importance of contextual factors, which may vary from one individual to another, and these may well include religious beliefs.

From the perspective of experimental phenomenology, however, it is essential to keep a focus on *experiences*, while abstaining from assumptions about the truth or falsehood of various religious beliefs (e.g., about God, eternal life, impermanence, or no-self). As argued by Brumett (2021) present-day mindfulness research partly fails to live up to this requirement, as it is partly enmeshed with Buddhist conceptualizations of the world, which are thought to represent “seeing things as they are.” Thompson (2020) makes a similar point when he argues against seeing meditation as a source of empirical knowledge:

“Buddhist meditation isn’t controlled experimentation. It guides people to have certain experiences and to interpret them in ways to conform to and confirm Buddhist doctrine. The claims that people make from having these experiences aren’t subject to independent peer review; they’re subject to assessment within the agreed-upon and unquestioned framework of the Buddhist soteriological path” (Thompson, 2020, p. 41).

It is essential here to be clear about the claims of experimental phenomenology and how these differ from claims about meditation as a form of scientific method. Experimental phenomenology is not about studying the experiences during meditation or other contemplative practices *as a source of knowledge about the world.* Experimental phenomenology relies on a systematic variation of phenomenological practices (including meditation and other contemplative practices) to establish *correlations between practices and experiences*, and in no way aspires to use experiences during these practices as a way of finding out truths about the world. The purpose of experimental phenomenology is to provide knowledge on practical and personalized ways of improving the life-quality of individuals, for example in the form of an improved health, and an increased presence, concentration, empathy, compassion, love, or wisdom.

## Some Examples From the Variety of Contemplative Practices

In the foregoing section, the conceptual framework of experimental phenomenology was described, and some illustrations were given of how it can be applied to the study of contemplative practices. The present section turns the perspective around and starts from the other direction. That is, it starts from two large areas of the human existence that may be made a focus of contemplation and gives examples of how aspects of these areas can be approached by means of experimental phenomenology. The two large areas that are chosen are (1) the contemplation of our being-in-the-world; and (2) the contemplation of being-with-others, or in other words, interpersonal relations.

### The Existential Contemplation of Being-in-the-World

Existential issues have had a central role in the writings of many philosophers and psychologists, and perhaps most notably among thinkers belonging to the so-called existentialist school (e.g., Kierkegaard, 1844; Sartre, 1938; Yalom, 1980). Existentialism involves the contemplation of specific themes in the life of human beings, such as (1) the inevitability of our death; (2) our basic aloneness as individuals, in the sense that no one else can share our most personal experiences; (3) the freedom we have in choosing how to live, and in making decisions and taking responsibility for our actions, as well as the anxiety and guilt that is associated with this kind of freedom; and (4) our having to find meaning in a world where there is no intrinsic meaning (e.g., Yalom, 1980).

These themes may represent important issues in the life of human beings. At the same time existentialism often appears to be biased toward those aspects of life that are associated with feelings such as guilt and anxiety (e.g., Kierkegaard, 1844), disgust (Sartre, 1938) and absurdity (Camus, 1955). There is less focus on the wonder of being, and on aspects of life that are associated with joy, love, and gratitude.

There are, however, other philosophers and religious thinkers, as well as psychologists (e.g., Seligman and Csikszentmihalyi, 2000), who have focused more on the joyous aspects of being-in-the-world. For example, the importance of cultivating gratitude has been a theme in many religions, including Hinduism, Buddhism, Judaism, Christianity, and Islam, and is also focused on by some contemporary psychologists and philosophers (for an overview, see Rushdy, 2020).

#### Gratitude and Gratefulness

Psychological research starting with Emmons and McCullough (2003) suggests that the practicing of gratitude may have effects on subjective wellbeing, although the results of meta-analyses (e.g., Dickens, 2017) indicate that the effects of this kind of practice tend to be rather modest. Here it is worth noting that the practices that were studied in this research are not primarily of a phenomenological kind. The participants were simply instructed to write down things for which they were grateful, on a daily or weekly basis. In terms of the verbal instructions, this may be seen as a behavioral practice, rather than a phenomenological one.

What, then, could a phenomenological practice look like in this area? To take an example, let us return to the contemplative practice that was described in the very first passage of the present paper: A person who “goes to the forest to spend time in contemplation of nature.” Suppose we would want to study this practice by means of an experimental-phenomenological design, to compare a gratitude practice with a simple form of mindfulness practice. This would require the development of specific instructions that are varied in terms of both attention and attitude. For example, the direction of attention in the mindfulness condition might be varied by means of formulations such as: “Note your specific perspective on what is in front of you,” “Note the forms and colors in your visual field,” “Note the sounds around you,” “Note any smells,” “Note the wind, the air you breathe, and your own breathing,” “Note your body situated in these surroundings,” “Note your own thoughts as you stand here in the forest,” etc. To change from mindfulness to an attitude of gratitude, the “note” part of the instructions may be changed, for example, into “thanks for” or some similar formulation. The primary research question here would be if a grateful attitude has other effects on the participants’ experience than a “noting” attitude.

Another issue that may also be addressed by means of experimental phenomenology is if gratitude can only be felt in relation to a person, or whether it can also be felt in relation to some larger impersonal context that one is part of. Gratitude in relation to other persons is generally referred to as interpersonal gratitude. A possible example of a more impersonal form of gratitude is what Nakhnikian (1961) referred to as “cosmic gratitude,” illustrating it by the feelings one can have on “contemplating the vast heavens on a starry night,” and then moving from awe, wonder, and reverence at that sight to a sense of cosmic gratitude (p. 158).

Formulations such as these have led to a discussion among philosophers of whether it is possible to experience gratitude without directing this gratitude to someone. Some religious philosophers (e.g., Roberts, 2014) have argued that gratitude is by necessity always felt to a benefactor of some kind, and if this benefactor is not another person it must be God. Against this, non-religious philosophers such as Solomon (2007) have argued that cosmic gratitude should not be understood on the same model as interpersonal gratitude. Whereas interpersonal gratitude is always directed to a person as benefactor, and felt for a specific gift of some kind, cosmic gratitude is something quite different, which is felt “for one’s whole life” (Solomon, 2007, p. 270).

According to Solomon (2007), cosmic gratitude is an essential element of spirituality, which he defines as “a meta-emotion that transcends the merely personal by taking into account our larger (or more modest) place in the universe” (p. 268). Solomon (2007) suggests various verbal formulations to describe the nature of such an all-embracing gratitude. In one formulation, he describes cosmic gratitude as “a way of putting one’s life in perspective” (p. 269) while “being properly humble about one’s own modest place in the world.” In another passage he describes it as a capacity of “expanding one’s perspective” so that we come to “appreciate the beauty of the whole” while at the same time being “absorbed in our own limited projects and passions” (p. 270). Common to these formulations is that gratitude is felt for our life, seen as a part of something larger (the world, or universe, or “the whole”).

As pointed out by an independent reviewer, this is an issue that would be possible to study by comparing the practice of gratitude with and without its being directed to a personal (divine) benefactor. In terms of experimental phenomenology, this may be done by developing and comparing two distinct forms of phenomenological practices, where the objects of attention are the same, but the attitude is formulated in personal terms in one case (e.g., by expressing gratitude to God), and in impersonal terms in the other. Again, it would be important in the first stage of such a study to use an open response format in asking about the participants’ experiences, together with qualitative analyses of their reports. One possible result here is that the effects may differ between different subgroups of participants; for at least some of them the religiously framed instructions may be felt to have the most beneficial effects.

Some philosophers have suggested that we should use different words to distinguish between the gratitude we feel to other people and the cosmic kind of gratitude. Walker (1980–1981), for example, suggested that we should differentiate between gratefulness and gratitude. Gratitude, as he sees it, is what we express to another person who has given us something, whereas gratefulness need not be directed to another person. Still, even gratefulness is associated with a desire to *give* something in return; in fact, “this desire is the distinctive mark of gratefulness” (Walker, 1980–1981, p. 49). And when there is no specific person to thank, our gratefulness may be expressed in the form of a more generalized generosity and good will toward others.

Here it may be added that, if gratefulness is felt toward the world or “the whole,” as suggested by Solomon (2007), such generosity need not be expressed only to other persons but may also involve a general motivation to care for animals, plants, and other things around us, including the larger world that we are part of. For example, we may think of this “cosmic gratefulness” as something that is felt in relation to all good things given to us in life, and that is expressed in a will to give good things back to the world. The latter would implicate a desire to consciously shape our life by means of an ethical contemplation of gifts and gratefulness. One role for experimental phenomenology here would be to explore various phenomenological practices focused on the experience and expression of such gratefulness as to their effects also on people’s behavior in relation to other individuals.

Importantly, this kind of gratefulness does not imply any indiscriminate positive feelings to everything. For example, if some processes (e.g., environmental pollution, or global climate change) threaten to destroy the beauty of the whole, or parts of it, a grateful attitude of this kind would call for a selective shift to another attitude. If gratefulness implies caring for the world, it also means to find effective ways of working against anything that threatens to destroy it.

This points to some of the limitations but also to some of the unexplored potentials of experimental phenomenology. Experimental phenomenology is limited to focusing on people’s experiences, but it is difficult to handle real physical threats without behavioral and political change. Still, experimental phenomenology may have a role also in this context if it can help us to understand how changes in the regulation of attention and attitudes can contribute to behavioral change, and how the development of phenomenological practices can contribute to behavioral and political change.

### Contemplative Interpersonal Relating

Up to now the discussion has focused primarily on different aspects of the relation between the individual person and the world. Almost nothing has been said about interpersonal relations. Interpersonal relations represent a complex field, with many different aspects that can be focused on in contemplative practices.

Experimental phenomenology is not a one-person psychology but can also be developed along interpersonal lines. One example is the above-mentioned study by Langer et al. (2009) of orchestral musicians where the results showed that the audience preferred the music that was created in a mindful state over music that was created by musicians who were merely trying to recreate a past performance. Here mindfulness instructions to one group of persons (the musicians) were found to have effects on the experiences of another group of persons (the audience).

Another basic aspect of interpersonal relations is the ability of one person to take the perspective of another person, which represents a basic aspect of empathy (e.g., Davis, 1994). Empathy exists on many levels, from the most basic fact that we naturally tend to perceive other persons as experiencing, thinking, feeling, willing creatures like ourselves (Husserl, 1938/1970), to our ability to develop a more fine-tuned empathic understanding of another person’s more specific experiences, thoughts, feelings, and motives (e.g., Kohut, 1959).

Empathy implicates mindfulness to another person and cannot be reduced merely to positive response and a pretended interest. Langer et al. (2012) illustrated this in an experimental study where they compared two groups of adults who were to interview children. One group of adults were instructed to interview the children in a mindful way; their instruction was to notice what the child’s voice and body language could say about the child’s feelings, and to see if these things varied or remained the same during the interview. Another group of participants received the “mindless” instruction to pretend to be interested in what the child said during the interview and to be positive in everything they said to the child. The results showed that the children preferred to interact with the mindful adults, and that they tended to devalue themselves following the mindless but positively staged interaction.

Another important aspect of interpersonal relations concerns more intimate forms of giving and receiving. Betty Martin is an American chiropractor who has developed a program called *The wheel of consent* (Martin, 2021). Although this program does not make use of words such as “mindful” or “contemplative,” it involves a whole series of phenomenological practices of a contemplative kind, focused on touching, and designed to develop the skills of receiving and giving, with a focus on consent. In view of the difficulties that people may have in relating to each other in an expressive and yet respectful and empathic manner, and in view of the violations of consent that do occur in such contexts, this is probably an area of great potential for developing the quality of interpersonal relations.

Apart from dealing with an important area of interpersonal relating, Martin’s program is of clear interest from an experimental phenomenological perspective. The word “experimental” figures prominently in her teaching. The following quotation captures something essential about the program: “The reward is in the practice itself, and what makes any practice engaging is not the doing you do but the quality of attention you bring to it. With this practice everything is an experiment.” (Martin, 2021, p. 5). In other words, there are several reasons why it is of interest to choose this specific program as an example here. It is also of interest because it represents an example of a set of newly constructed contemplative practices, and serves as an illustration of how such practices are continually constructed among various practitioners on the basis of a kind of informal experimental phenomenology.

#### Betty Martin’s Wheel of Consent

This is a program based on three lessons and five labs, where each part builds on the previous ones and are to be followed in the given order. This section gives a brief description of the three first lessons. In the first lesson, the participants are trained in the mindful touching of ordinary things, with a focus on exploration and the pleasure of touching. In the two following lessons they are trained in mindful interpersonal touching (i.e., the touching of another person’s hand), again with a focus on exploration and pleasure. Because this involves two persons, it becomes more complicated and raises issues of personal limits and consent. This part of the training involves a contemplative approach to desire, limits, and consent, where the participants are instructed (1) to slow down and contemplate both their own desires and their limits regarding what they are comfortable with in interpersonal touching, and (2) to train interpersonal skills such as the respectful expression of desire, and the clear expression of personal limits in the interpersonal give-and-take.

##### Lesson One: Waking up the Hands

The first lesson of Martin’s (2021) program is called “Waking up the hands.” Here she instructs the participants to train in touching ordinary things, such as a cushion or a pen. An attitude of curiosity is encouraged. The participants are instructed to bring their attention to the hands, and to explore the object chosen to notice everything about it (its weight, shape, texture, etc.), and to feel the pleasure in touching it. Just as in different varieties of meditation, the participants are instructed to make this choice again and again when they get distracted. They are also instructed to go slow, because the slower they move their hands the more they can feel, and the more there is to notice. It is emphasized that there is no goal, other than focusing on how it feels in the present moment to touch this object.

From an experimental phenomenological perspective, the overall attitude is explorative. The focus of attention is first on the details of the object that is touched, and then shifts increasingly to the sensations in the hands and to the feelings of pleasure in touching the object. Importantly, the attitude involved also concerns the tempo of the touching (i.e., to proceed slowly). Although this may seem to be a simple exercise, Martin (2021) gives an overview of the difficulties many people have with it. She describes large individual differences in how much practice it takes for participants to get access to a relaxed state of pleasure in the process, and to an experience of their hands as a source of pleasure. At the same time, she regards this first lesson as foundational for the rest of the program. Practicing “waking up the hands” is said to have the potential to contribute to the development of an increased quality of the person’s touch also in interpersonal contexts.

##### Lesson Two: The Take/Allow Dynamic

In the second lesson the object to be explored by touch is not a thing but another person’s hand. In other words, the situation now involves two persons instead of one person and a thing. These two persons have an equal status in the process, not only because they change roles after half the time, but also because *consent* is central to the exercise. Here several complications arise.

First, because the exploration is now directed at another person and not at a thing, the second person must *allow* the first person to do this. And is the second person really *willing* to do this? Here consent enters the scene. An essential part of the exercise is that the second person has the right to refuse, and to set limits, and to tell the first person to stop if something does not feel right.

From the perspective of experimental phenomenology it is interesting to note how precise Martin (2021) is in her instructions to the participants. Here the first person is instructed to ask, “May I feel your hand,” with exactly these words, and to wait for an answer. The second person is instructed to pause and “consider whether this is a gift you can give with a full heart” (p. 86), and not to say yes until they hear “an inner yes” and then to respond with the exact words “Yes, you may.” If the second person is not willing, the instruction is to say “No, not today.” If he/she is willing only within certain limits, the instruction is to clearly specify these (e.g., “only my right hand, not the left”). And if something does not feel right during the process, the instruction is to express this clearly. It is essential here that the person whose hand is to be touched feels that he/she *has a choice* throughout the exercise.

One main difficulty during this exercise is that the first person may slip into a giving attitude (e.g., by caressing or massaging the second person’s hand). But what is to be trained here is not the skills of giving, but an attitude of curiosity about the other person’s hand: “Respect any limits your partner has set, and then feel their hand. That’s it.” (Martin, 2021, p. 88). In other words, it is equally essential both to respect any limits that the other person has, and to keep an attitude of curiosity about the other’s hand and how it feels to touch it. The purpose is to *explore* another person’s hand in the same way that the external thing was explored in lesson one – that is, with full attention to the other’s hand, to notice everything about it as well as the pleasure that goes with this. And again, the instruction is to gently bring attention back when being distracted, and to go slowly to notice as much as possible about the other’s hand and how it feels.

As Martin (2021) puts it, there is a gift involved in this exercise, but it is not the actively touching partner who is the giver. The active partner, in fact, *receives* a gift by being allowed to explore and feel the other person’s hand. Accordingly, when the participants are instructed to end the exercise by looking each other in the eyes and say, “Thank you” and “You are welcome,” it is the actively touching partner who says thank you.

Many people have difficulties finding this attitude – an attitude which Martin refers to as *taking* and which involves a combination of two things: putting one’s own desires first, and respecting the limits of the giver (Martin, 2021, p. 97). A common reason for these difficulties, she says, is that putting one’s own desires first is often assumed to be somehow unkind or hurtful to the other person. In Martin’s experience, however, the effects of the practice are often the other way around. When the person finally finds this attitude (“this is for me”) it tends to produce a notable change in the touching, which now slows down and becomes more relaxed, explorative, and sensual, while taking in “a tremendous amount of information” (Martin, 2021, p. 93). And this may, in fact, be experienced as quite pleasurable also for the person who is being touched: “I have had many people say this was the best touch they’ve ever felt.” (Martin, 2021, p. 102).

The two persons in this exercise are referred to as the “taking” and the “allowing” partner, and the interpersonal process is called the *take/allow dynamic* (Martin, 2021). “Taking” is a word with many meanings, but here it means taking the gift that is provided by the allowing partner – that is, taking the opportunity to explore that person’s hand with full curiosity, and the joy and pleasure that comes with this, while fully respecting that person’s limits. This take/allow dynamic is contrasted to another interpersonal process which Martin (2021) refers to as the *serve/accept dynamic*. This is the subject for the third lesson. Here the direction of the gift is reversed – now it is the active partner who provides the gift.

##### Lesson Three: The Serve/Accept Dynamic

In the third lesson the question asked is “How would you like me to touch your hands?” This means that the active person now takes a *serving* attitude. The second person is to pause for a while to contemplate how he or she really wants to be touched, and then to respond with a request of the form “I would like… will you do that?” The person may, for example, come up with a request such as, “I would like you to softly caress my palm with your fingertips. Will you do that?” (Martin, 2021, p.107).

Here the serving partner may follow up with further questions to become clear about exactly how the person wants to be touched. The instruction is: “Ask anything you need to clarify. Do they mean soft, light, firm, slow, deep? Gently ask for the information you need so you can give them exactly what they want.” When it is fully clear what the other person wants, the serving person is to pause and contemplate whether this is something that he/she really can give with a full heart. If it is not, the request may need to be negotiated. After that the serving person says, “Yes, I will,” and the process starts (Martin, 2021, p. 107–108).

Again, the two participants have an equal status in the process, not only by changing roles after half the time, but also because consent is of central importance in the process. The serving partner needs to feel that the request is within his or her limits, and the accepting partner must feel that the touch that is given is in accordance with their agreement. The serving partner is to do only what was asked for and nothing else, as well as to check with the receiving partner to make sure that this is what they had in mind.

The receiving partner is instructed not to pretend to like anything other than what was asked for, and to ask for changes at any time to make sure that they receive exactly what they want. “When you receive exactly the touch you asked for, something lands in your heart. You notice, perhaps for the first time, that this really is for you” (Martin, 2021, p. 110).

Just as in session 2, the skills that are trained are both contemplative (searching within oneself to find out about one’s desires and limits, as well as pondering the other person’s desires and limits and respecting these) and communicative (expressing one’s desires and limits, and eliciting feedback from the other person). The sessions are similar also in that they both involve a training in how to differentiate between the attitudes of receiving (“this is for me”) and giving (“this is for them”); at the same time, however, they represent a clear contrast in how these two attitudes combine with active touching and with being touched. In session 2, *receiving* is combined with doing whereas giving is combined with being done to, whereas in session 3 *giving* is combined with doing whereas receiving is combined with being done to and – together these four combinations represent the “four quadrants” in Martin’s model of the Wheel of Consent. A basic principle in her thinking is that the two attitudes of receiving and giving need to be taken apart and trained separately in these two different contexts: “In order to experience them, we have to separate them. We have to experience them one at a time, undiluted by the other” (p. 35).

To summarize, Martin’s (2021) *Wheel of Consent* is an interesting example of a complex program of phenomenological practices. Although it makes no use of the terms “mindfulness” or “contemplative” it seems to have many typical characteristics of a phenomenological practice of a contemplative kind: regulation of attention (with a focus on touching) and attitudes (practicing an explorative attitude and differentiating between the attitudes of receiving and giving), precise verbal instructions, an emphasis on going slow, and clear elements of contemplation (of desire, limits, and consent). It also shares many characteristics with the kind of contemplative practice as a life project that is described by Komjathy (2017), such as a commitment to practice for the purpose of personal development. Martin (2021), for example, carefully describes the difficulties that many people have with the seemingly simple phenomenological practices that are involved, and she also describes the skills of touching, receiving and giving as something that may continue to develop all through a person’s life.

Martin’s (2021) *Wheel of Consent* can be seen as an example of how an informal variety of experimental phenomenology may lead to the development of a new complex program of phenomenological practices. It remains, however, to study these phenomenological practices as part of a more research-oriented experimental phenomenology; this would require a systematic variation of the attention and attitude that characterize these practices. It would also be interesting to study the long-term effects of the program; this would require the use of additional methods such as longitudinal designs and measures both of commitment and of the kind of skills that are involved.

## Discussion

Although some contemplative practices are very old, the field of Contemplation Studies is still young. Among other things, this means that there are no agreed-upon definitions of central concepts such as contemplative practices and contemplative experiences. This presents a difficulty for discussions in this area. On the other hand, it may be argued that it is a good thing to be open regarding such definitions at the present stage of research (cf. Komjathy, 2017, p. 69).

The present paper started from a working definition of contemplative practices as practices that are engaged in for the contemplative experiences that they afford. A paradigm example that was suggested is a person who goes into the forest to contemplate nature. In this case there need be no other purposes involved than the contemplative experience itself. Because of the beneficial effects that have been experienced as part of such experiences, however, people in many different cultures and as part of many different traditions (religious and secular) have started to cultivate specific contemplative practices by practicing these on a regular basis for the purpose of higher-order goals such as improved health, attentiveness, concentration, presence, awareness, compassion, and wisdom. This involves a commitment to practice for the purpose of some form of personal development. Komjathy (2017) has accordingly described a contemplative life path, characterized by three elements: commitment to practice, critical subjectivity, and character development.

A rigorous scientific study of contemplative practices requires a comprehensive approach in terms of an integrative conceptual framework. The purpose of the present paper was to describe the basic characteristics of an approach called experimental phenomenology (Lundh, 2020) and to discuss its potential to contribute to the development of such an integrative framework. Experimental phenomenology is defined here as an investigation of phenomenological practices and their effects on experience. Phenomenological practices involve an intentional variation of experience by changes in the direction of attention and the choice of attitude, typically as guided by some form of verbal instructions and self-instructions. Experimental phenomenology rests on a systematic variation of such phenomenological practices for the purpose of establishing causal connections, or at least correlations, between various practices and the resulting experiences.

A basic assumption in the present paper has been that contemplative practices are a subcategory of phenomenological practices. To the extent that this is true, experimental phenomenology is directly relevant both for the *development* of new contemplative practices and for the *study* of existing ones. As to the development of new contemplative practices, the hypothesis is that *an informal variety of experimental phenomenology* has been used by creative innovators during all ages when they have developed new practices in this area. That is, when new contemplative practices have been developed in different cultures and traditions, this has probably been done based on observations of correlations between various kinds of practices and the resulting experiences. Martin’s (2021) Wheel of Consent represents a recent program that has been developed by its originator over the years with the help of observations of this kind. A similar kind of informal experimental phenomenology may also be used by present-day clinicians to develop personalized health interventions. Two examples that have been discussed are a mindful driving practice (Lundh, 2020) and a mindful embodiment exercise (Lundh, 2021).

This kind of informal experimental phenomenology does not, however, qualify as a scientific method. To qualify as a scientific method experimental phenomenology needs to be applied to well-defined phenomenological practices in accordance with experimental designs, where the direction of attention and the choice of attitudes are varied systematically to find correlations between variations in the practice and variations in the resulting experiences. It is possible that this kind of research may lead not only to a more systematic theoretical knowledge of general principles that underly contemplative practices, but also to the development of new practices based on this understanding.

This may be seen as analogous to the various kinds of technologies (e.g., learning to control the fire) that were developed by early humans in the form of a practical knowhow long before they had any theoretical knowledge about the combustion processes that are involved. Nowadays, however, the process within the field of technology often runs in the opposite directions; theoretical knowledge about quantum physics, for example, has made possible the development of many new technologies such as computers and mobile phones. The question may be asked if a similar kind of development can occur with contemplative practices: Is it possible that a theoretical knowledge about the general principles underlying phenomenological practices and their effects can eventually lead to the development of new contemplative practices with a superior impact on human well-being and personal development?

One limitation of experimental phenomenology is that it primarily addresses single contemplative practices and their resultant experiences. This only represents one of the three characteristics which Komjathy (2017) sees as typical of contemplative practice as a life project – the other two being commitment to practice, and goals of character development. The study of contemplative practice as a life project, in addition to experimental phenomenology, would require other methods such as, for example, longitudinal research designs and ways of measuring regularity of practice and various aspects of personal development.

A final comment on the term *experimental* phenomenology: To avoid confusion here, it should be remembered that the word “experimental” has at least two important but different meanings, which are both relevant to experimental phenomenology. First, from a scientific perspective, controlled experiments are invaluable tools in our efforts to understand causal relationships. Conducting experimental research means to study the effect of various interventions, while trying to control other variables that may affect the outcome. Experimental phenomenology makes it possible to study the most immediate effects of variations in a contemplative practice by means of a systematic variation of the instructions given about direction of attention and choice of attitude.

The second meaning of “experimental” has less to do with science; to act “experimentally” here simply means to test something new out of curiosity or in a spirit of creativity. If this kind of experimental approach is directed at our way of paying attention to things, or to the attitude we take to things, and is followed by an observation of the experiences that follow, then we are engaging in an informal variety of experimental phenomenology. This kind of informal experimentation probably lies at the basis of a lot of the contemplative and other phenomenological practices that are around today. Further, this informal variety of experimenting represents an important first stage of exploration before we eventually engage in more controlled experiments – it may, for example, lead to the formulation of hypotheses that can be tested more rigorously in scientifically controlled studies.

A working assumption of experimental phenomenology as defined here is that this approach represents a new perspective on contemplative practices that may contribute to the development of this area of study. Although the present paper has applied this framework to the understanding of a variety of contemplative practices, this still represents a rather limited sample of such practices. There exist many other contemplative practices, and the question remains whether they can all be approached in terms of experimental phenomenology (i.e., in terms of the regulation of attention and attitudes, as defined by a set of verbal instructions). This should be seen as a hypothesis that, like all scientific hypotheses, needs to be subjected to continued critical testing and that will be strengthened to the extent that it survives such testing.

## Data Availability Statement

The original contributions presented in the study are included in the article/supplementary material, further inquiries can be directed to the corresponding author.

## Author Contributions

L-GL wrote the entire manuscript.

## Conflict of Interest

The author declares that the research was conducted in the absence of any commercial or financial relationships that could be construed as a potential conflict of interest.

## Publisher’s Note

All claims expressed in this article are solely those of the authors and do not necessarily represent those of their affiliated organizations, or those of the publisher, the editors and the reviewers. Any product that may be evaluated in this article, or claim that may be made by its manufacturer, is not guaranteed or endorsed by the publisher.

## References

[B1] Albertazzi L. (2019). Experimental phenomenology: what it is and what it is not. *Synthese* 198 S2191–S2212. 10.1007/s11229-019-02209-6

[B2] Alexander F. M. (1932). *The Use Of The Self.* London: Orion Books.

[B3] Bergman L. R. Lundh L. G. (2015). Introduction: the person-oriented approach: roots and roads to the future. *J. Per. Oriented Res.* 1 1–6. 10.17505/jpor.2015.01

[B4] Bishop S. R. Lau M. Shapiro S. Carlson L. Anderson N. D. Carmody J. (2004). Mindfulness: a proposed operational definition. *Clin. Psychol. Sci. Pract.* 11 230–241. 10.1093/clipsy.bph077

[B5] Brumett A. (2021). The vagueness of clarity: metaphysical and epistemic truth claims in the empirical study of mindfulness practices. *Mindfulness* 12 2132–2140. 10.1007/s12671-021-01683-9

[B6] Camus A. (1955). *The Myth Of Sisyphus And Other Essays.* New York: Alfred A. Knopf.

[B7] Cramer H. Ward L. Steel A. Lauche R. Dobos G. Zhang Y. (2016). Prevalence, patterns, and predictors of yoga use: results of a U.S. nationally representative survey. *Am. J. Prev. Med.* 50 230–235. 10.1016/j.amepre.2015.07.037 26497261

[B8] Davis M. H. (1994). *Empathy. A social psychological approach.* Madison, Wis: Brown & Benchmark.

[B9] Dahl C. J. Lutz A. Davidson R. J. (2015). Reconstructing and deconstructing the self: cognitive mechanisms in meditation practice. *Trends Cogn. Sci.* 19, 515–523. 10.1016/j.tics.2015.07.001 26231761 PMC4595910

[B10] Dickens L. R. (2017). Using gratitude to promote positive change: a series of meta-analyses investigating the effectiveness of gratitude interventions. *Basic Appl. Soc. Psychol.* 39 193–208. 10.1080/01973533.2017.1323638

[B11] Elkins G. R. Barabasz A. Council J. R. Spiegel D. (2015). Advancing research and practice: the revised APA Division 30 definition of hypnosis. *Int. J. Clin. Exp. Hypn.* 63 1–9. 10.1080/00207144.2014.961870 25365125

[B12] Emmons R. McCullough M. E. (2003). Counting blessings versus burdens: an experimental investigation of gratitude and subjective well-being in daily life. *J. Pers. Soc. Psychol.* 84 377–389. 12585811 10.1037//0022-3514.84.2.377

[B13] Feldenkrais M. (1972). *Awareness through Movement.* New York: HarperCollins.

[B14] Freud S. (1916). “Introductory lectures to psychoanalysis,” in *Standard edition of the complete psychological works of Sigmund Freud*, ed. Strachey J. (London: Hogarth Press), 3–239.

[B15] Gendlin E. T. (1978). *Focusing.* New York: Bantam Books.

[B16] Husserl E. (1938/1970). *The Crisis Of The European Sciences And Transcendental Phenomenology. [Originally published in German as Die Krisis der europäischen Wissenschaften und die transzendentale Phänomenologie. Martinus Nijhoff.].* Evanston: Northwestern University Press.

[B17] Ihde D. (1977). *Experimental phenomenology. An introduction.* New York: Putnam.

[B18] Ihde D. (2012). *Experimental phenomenology. Second edition.* New York: University of New York Press.

[B19] Kabat-Zinn J. (2013). *Full catastrophe living: using the wisdom of your body and mind to face stress, pain and illness. Revised and updated edition.* New York: Bantam Books.

[B20] Kierkegaard S. (1844). *The concept of anxiety.* Princeton, New Jersey: Princeton University Press

[B21] Kohut H. (1959). Introspection, empathy, and psychoanalysis. *J. Am. Psychoanal. Assoc.* 7 459–483. 10.1177/000306515900700304 13672863

[B22] Komjathy L. (2017). *Introducing Contemplative Studies.* Hoboken: John Wiley & Sons.

[B23] Langer E. J. (2014). *Mindfulness. 25th Anniversary Edition.* Boston: Da Capo Press.

[B24] Langer E. J. Cohen M. Djikic M. (2012). Mindfulness as a psychological attractor: the effect on children. *J. Appl. Soc. Psychol.* 42 1114–1122. 10.1111/j.1559-1816.2011.00879.x

[B25] Langer E. Russel T. Eisenkraft N. (2009). Orchestral performance and the footprint of mindfulness. *Psychol. Music* 37 125–136. 10.1177/0305735607086053

[B26] Lindahl J. R. Fisher N. E. Cooper D. J. Rosen R. K. Britton W. B. (2017). The varieties of contemplative experience: a mixed-methods study of meditation-related challenges in Western Buddhists. *PLoS One* 12:e0176239. 10.1371/journal.pone.0176239 28542181 PMC5443484

[B27] Luberto C. M. Shinday N. Song R. Philpotts L. L. Park E. R. Fricchione G. L. (2018). A systematic review and meta-analysis of the effects of meditation on empathy, compassion, and prosocial behaviors. *Mindfulness* 9 708–724. 10.1007/s12671-017-0841-8 30100929 PMC6081743

[B28] Lundh L. G. (2015). The person as a focus for research – The contributions of Windelband, Stern, Allport, Lamiell and Magnusson. *J. Pers. Oriented Res.* 1 15–33. 10.17505/jpor.2015.03

[B29] Lundh L. G. (2020). Experimental phenomenology in mindfulness research. *Mindfulness* 11 493–506. 10.1007/s12671-019-01274-9

[B30] Lundh L. G. (2021). Experimental phenomenology as personalized health intervention. A case illustration.

[B31] Martin B. (2021). *The art of receiving and giving. The wheel of consent.* Oregon: Luminare Press.

[B32] Martiny K. M. Toro J. Høffding S. (2021). Framing a phenomenological mixed method: from inspiration to guidance. *Front. Psychol.* 12:602081. 10.3389/fpsyg.2021.602081 33746828 PMC7966507

[B33] Merriam-Webster Dictionary (n.d.). *Contemplation.* Available Online at: https://www.merriam-webster.com/dictionary/contemplation#examples (accessed December 13, 2021)

[B34] Nakhnikian G. (1961). “On the cognitive import of certain conscious states,” in *Religious Experience and Truth: a Symposium*, ed. Hook S. (New York: New York University Press).

[B35] Petitmengin C. Van Beek M. Bitbol M. Nissou J. M. Roepstorff A. (2017). What is it like to meditate? Methods and issues for a micro-phenomenological description of meditative experience. *J. Conscious. Stud.* 24 170–198.

[B36] Roberts R. C. (2014). Cosmic gratitude. *Eur. J. Philos. Relig.* 6 65–83.

[B37] Rose K. (2016). *Yoga, Meditation, and Mysticism: Contemplative Universals and Meditative Landmarks*. London: Bloomsbury Academic.

[B38] Rushdy A. H. A. (2020). *Philosophies Of Gratitude.* New York, NY: Oxford University Press, 10.1093/oso/9780197526866.001.0001

[B39] Ryan R. M. Deci E. L. (2000). Intrinsic and extrinsic motivations: classic definitions and new directions. *Contemp. Educ. Psychol.* 25 54–67. 10.1006/ceps.1999.1020 10620381

[B40] Salzberg S. (2002). *Lovingkindness: the Revolutionary Art Of Happiness.* Boulder, Colorado: Shambhala.

[B41] Sartre J. P. (1938). *Nausea.* London, England: Penguin Classics

[B42] Seligman M. E. P. Csikszentmihalyi M. (2000). Positive psychology: an introduction. *Am. Psychol.* 55 5–14. 10.1037/0003-066x.55.1.5 11392865

[B43] Shapiro S. L. Carlson L. E. (2017). *The Art And Science Of Mindfulness: integrating Mindfulness Into Psychology And The Helping Professions.* Washington, DC: American Psychological Association.

[B44] Solomon R. C. (2007). *True to our feelings: what our emotions are really telling us.* Oxford: Oxford University Press.

[B45] Sumantry D. Stewart K. E. (2021). Meditation, mindfulness, and attention: a meta-analysis. *Mindfulness* 12 1332–1349. 10.1007/s12671-021-01593-w

[B46] Thompson E. (2020). *Why I am not a Buddhist.* England: Yale University Press.

[B47] Vieten C. Wahbeh H. Cahn B. R. MacLean K. Estrada M. Mills P. (2018). Future directions in meditation research: recommendations for expanding the field of contemplative science. *PLoS One* 13:e0205740. 10.1371/journal.pone.0205740 30403693 PMC6221271

[B48] Walker A. D. M. (1980–1981). Gratefulness and gratitude. *Proc. Aristotelean Soc. New Ser.* 81 39–55.

[B49] Wang C. Li K. Gaylord S. (2019). Prevalence, patterns, and predictors of meditation use among U.S. children: results from the National Health Interview Survey. *Complement. Ther. Med.* 43 271–276. 10.1016/j.ctim.2019.02.004 30935542 PMC6502253

[B50] Yakobi O. Smilert D. Danckert J. (2021). The effects of mindfulness meditation on attention, executive control and working memory in healthy adults: a meta-analysis of randomized controlled trials. *Cognit. Ther. Res.* 45 543–560. 10.1007/s10608-020-10177-2

[B51] Yalom I. D. (1980). *Existential psychotherapy.* New York: Basic Books.

